# Identifying the barriers and enablers in the implementation of the New Zealand and Australian Antenatal Corticosteroid Clinical Practice Guidelines

**DOI:** 10.1186/s12913-016-1858-8

**Published:** 2016-10-28

**Authors:** E. L. Mc Goldrick, T Crawford, J. A. Brown, K. M. Groom, C. A. Crowther

**Affiliations:** 1 Liggins Institute, The University of Auckland, 85 Park Road, Grafton, Auckland, 1023 New Zealand; 2 Department of Obstetrics and Gynaecology, The University of Auckland, Auckland, New Zealand; 3 National Womens Health, 2 Park Road, Auckland, 1023 New Zealand; 4 The Liggins Institute, The University of Auckland, Building 503, Level 2, 85 Park Road, Auckland Private Bag 92019, Auckland, 1142 New Zealand

**Keywords:** Implementation, Clinical practice guidelines, Antenatal corticosteroids, Theoretical domains framework

## Abstract

**Background:**

The ineffective implementation of evidence based practice guidelines can mean that the best health outcomes are not achieved. This study examined the barriers and enablers to the uptake and implementation of the new bi-national (Australia and New Zealand) antenatal corticosteroid clinical practice guidelines among health professionals, using the Theoretical Domains Framework.

**Methods:**

Semi-structured interviews or online questionnaires were conducted across four health professional groups and three district health boards in Auckland, New Zealand. The questions were constructed to reflect the 14 behavioural domains from the Theoretical Domains Framework. Relevant domains were identified by the presence of conflicting beliefs within a domain; the frequency of beliefs; and the likely strength of the impact of a belief on the behaviour using thematic analysis. The influence of health professional group and organisation on the different barriers and enablers identified were explored.

**Results:**

Seventy-three health professionals completed either a semi-structured interview (*n* = 35) or on-line questionnaire (*n* = 38). Seven behavioural domains were identified as overarching enablers: belief about consequences; knowledge; social influences; environmental context and resource; belief about capabilities; social professional role and identity; and behavioural regulation. Five behavioural domains were identified as overarching barriers: environmental context and resources; knowledge; social influences; belief about consequences; and social professional role and identity. Differences in beliefs between individual health professional groups were identified within the domains: belief about consequences; social professional role and identity; and emotion. Organisational differences were identified within the domains: belief about consequences; social influences; and belief about capabilities.

**Conclusion:**

This study has identified some of the enablers and barriers to implementation of the New Zealand and Australian Antenatal Corticosteroid Clinical Practice Guidelines using the validated Theoretical Domains Framework, as perceived by health professionals. We have identified differences between individual health professional groups and organisations. The identification of these behavioural determinants can be used to enhance an implementation strategy, assist in the design of interventions to achieve improved implementation and facilitate process evaluations to understand why or how change interventions are effective.

**Electronic supplementary material:**

The online version of this article (doi:10.1186/s12913-016-1858-8) contains supplementary material, which is available to authorized users.

## Background

Limited information is known on the process of how and why clinicians change their practice and currently there is no standardised implementation strategy known to be completely effective in translating research findings into clinical practice [1–3]. Increasingly, global health organisations and governments are urging researchers to identify effective strategies to reduce barriers to the uptake of proven interventions and minimise evidence practice gaps [4, 5].

Barriers and enablers are determinants of healthcare practice that may prevent or facilitate improvements in practice [6]. Barriers can exist at multiple levels. The Cochrane Effective Practice and Organisation of Care Group classify barriers into nine categories (information management, clinical uncertainty, sense of competence, perceptions of liability, patient expectations, standards of practice, financial disincentives, administrative constraints and other) [7]. It has been argued that performing a comprehensive assessment of the barriers and enablers is key in developing an informed implementation strategy. Intervention strategies that are chosen to overcome the pre-identified barriers should subsequently improve practice as a result [1, 8]. It is believed that this will advance the current knowledge in understanding the causal mechanisms through which the intervention worked, and how the chosen intervention modified or enhanced the pre-identified barriers and enablers [3]. A Cochrane systematic review in 2010 recommended that future implementation studies must explicitly describe how barriers were identified and how overcoming these would form part of any implementation strategy [9].

Improving implementation of evidence-based practice by healthcare professionals often requires changing multiple behaviours of a number of individuals at many different levels [3]. Changing behaviour can be complex. The effectiveness of implementation strategies is sensitive to context [10] and the limited practical value of these strategies may be attributed to the atheoretical nature of many of them [8]. Applying psychological theories to the identification of barriers is more likely to identify opportunities and methods to develop a successful and targeted implementation strategy [11]. The Theoretical Domains Framework (TDF) [8] simplifies and integrates a plethora of behavioural change theories to assist researchers involved in evidence based practice implementation. The refined and validated framework contains a set of 14 behavioural domains that cover the main factors influencing individual practitioner behaviour and behaviour change [12]. The framework can be used to identify and explain why implementation of evidence based practice has not occurred and to design interventions to achieve improvements in implementation [8].

Clinical practice guidelines have been identified as an invaluable resource in attempting to assist practitioner and patient decisions about appropriate health care in the clinical environment [1, 2]. Despite the recognition that a single course of antenatal corticosteroids reduces death and major morbidity in preterm infants [13, 14], significant uncertainty still exists in their use in particular populations and circumstances [15, 16]. A new bi-national (New Zealand and Australia) clinical practice guideline entitled: “Antenatal corticosteroids given to women prior to birth to improve fetal, child and adult health”, has been developed with the aim of providing, practical evidence based guidance on best practice for clinical care in women prior to birth to improve fetal, infant, child and adult health [17]. Passive implementation of a clinical practice guideline is unlikely to ensure uptake and sustained use in clinical practice [18–21]. This study aimed to identify the overarching enablers and barriers to implementation of the new Clinical Practice Guideline prior to its release and highlight existing evidence practice gaps. The findings will help to identify if interventions can be generalisable or need to be tailored to individual health professional groups or sites.

## Methods

### Design

This, qualitative study was conducted among health professionals who care for pregnant women and/or infants born preterm, working within the three district health boards within Auckland, New Zealand. Semi-structured interviews or online questionnaires were designed using the Theoretical Domains Framework [8, 12].

### Setting

This study was conducted at four maternity hospitals in New Zealand. The four hospital sites represent three different district health boards within Auckland and serve a catchment population of 1,415,550 representing 32 % of the New Zealand population [22]. Approximately 39 % of babies born in maternity facilities in New Zealand, are born within these three district health boards [23]. National Women’s Health (Auckland City Hospital) provides level three neonatal intensive care to the Northland region, Central Auckland, West Auckland and North Auckland areas. The neonatal service at National Women’s Health receives 1,600 admissions a year, making it the largest newborn unit in Australasia. It receives babies from other New Zealand regions and cares for babies requiring neonatal surgery and other specialist services. Middlemore Hospital provides level three neonatal intensive care, predominantly to the South Auckland/Counties Manukau region as well as providing extra capacity at peak demand times for regional and national overflow. These level three units provide care to babies who need ventilation, are born at less than 30 weeks’ and/or require intensive care support. Waitemata District Health Board encompasses North Shore and Waitakere Hospitals which care for women with low or medium risk pregnancies and their Special Care Baby Units can care for babies from 32 weeks’ gestation onwards.

### Participants

The health professional groups recruited included obstetricians, midwives, neonatologists and paediatricians. A purposive sampling method was used to recruit junior and senior health professionals. Midwives and doctors who worked within the hospital and as private lead maternity carers were recruited, to capture the breadth of opinions reflective of the diverse health care environment. A lead maternity carer may be public or private, hospital or community based and is responsible for managing the woman’s care throughout pregnancy, birth and immediately postpartum.

Participants were identified in two ways. At each of the hospital sites a key person was identified who sent out an email to all health professionals on the hospital mailing list inviting health professionals to participate in the study. Participants demonstrated interest by replying to the email and then received a participant information sheet and consent form to complete. Secondly, on occasion health professional participants were approached directly by one of the researchers (EM) and invited to take part in the study. This study is nested within a randomised trial to assess different methods for identifying barriers and enablers to administration of antenatal corticosteroids (to be reported elsewhere). Consequently following completion of a signed consent form, participants were randomised to either a semi-structured interview or online questionnaire. Responses from both methods were analysed and reported together in this study. All information collected during the interview or questionnaire process was confidential and anonymised for analysis and reporting.

We aimed to recruit eight individuals per professional group (obstetrician, midwife, neonatologist or paediatrician) at each of the three district health boards to facilitate data saturation in the thematic analysis and to allow comparison across professional groups and institutions. Neonatologists and paediatricians were recruited and analysed together. They were defined as clinicians who look after the babies on the neonatal units across the three district health boards that participated in the study. During the remainder of the paper they are referred to as neonatologists.

Ethical approval was obtained by the University of Auckland Health Participants Ethics Committee (011193) and locality agreement was obtained at each of the respective sites.

### Materials

Questions were developed to cover the domains and their component constructs within the TDF [8, 12]. This resulted in each domain being linked to a set of questions (Additional file 1). The questions used in both the semi-structured interview and online questionnaire were the same and were piloted by members of the research group and by colleagues working within the professional groups (obstetricians, neonatologists and midwives) to minimise repetition and ensure clarity of the questions.

The questions explored different health professionals’ attitudes, beliefs, and knowledge about the prescription or administration of antenatal corticosteroids and identified possible reasons for any evidence practice gaps. Beliefs about clinical practice guidelines, the need for a bi-national (New Zealand and Australia) antenatal corticosteroid clinical practice guideline and how these guidelines could best be implemented were explored. Further analysis investigated the influence of individual health professional groups and health care organisations on the different barriers and enablers identified.

Demographic questions were included to capture participants’ age, ethnicity, current position, primary place of work and years of experience.

### Data collection

Each participant was given a unique identifying number which allowed all recordings and transcripts to be anonymised for the purpose of confidentiality. Only the researcher (EM) had knowledge of the participant names and their corresponding unique identifying numbers. This allowed analysis within and between different health professional groups.

The semi-structured interviews and online questionnaires were completed over an 8 month time period (April to November 2014) prior to the release of the guideline. No explicit time restraints were applied to either assessment method. Each semi-structured interview or online questionnaire was expected to take 20 min to complete.

### Interview procedures

Semi-structured, face to face interviews were conducted by a single researcher (EM); a PhD student with a medical background in obstetrics and gynaecology and training in interview skills. Interviews were conducted at a location convenient to the participant or in a quiet office on the hospital site. Interviews were audio-recorded and handwritten notes were taken.

### Questionnaire

The online questionnaires were developed on the web based system, Survey Monkey® [24], and were emailed to the participants preferred email to be completed at a time convenient to them. Participants were asked to insert their unique identifying number at the beginning of the questionnaire. If participants did not have access to a computer a paper version of the questionnaire was provided. A reminder via email or text message was sent to non-responders 2 weeks after the initial questionnaire had been sent.

### Data analysis

Audiotaped interviews were reviewed and transcribed verbatim. The authenticity of the transcripts where confirmed by a second author who selected four (11 %) transcripts at random to compare with the audio recordings (TC). Two of the authors (EM and TC) read all of the transcripts at least twice to familiarise themselves with the data. To increase rigour, these two reviewers independently coded the anonymised semi-structured interview transcripts and the open ended online questionnaire responses using the TDF. All transcripts were coded in Nvivo 8 software [25].

Initially a directed approach to content analysis [26] was used to classify responses into one or more of the 14 theoretical domains [8, 12]. The reviewers (EM and TC) met after the first two transcripts had been coded to compare results and any differences in coding within behavioural domains were resolved through discussion. If there were any uncertainties the other authors (CAC, JB, KG) reviewed the coding and agreement was reached. This process continued with the remaining transcripts with the two reviewers meeting weekly to compare results. Following this the two researchers (EM and TC) reviewed all of the responses coded within each behavioural domain and used thematic analysis [27] to generate a list of specific beliefs to represent the overarching statements or themes coded within each behavioural domain [28].

The two reviewers (EM and TC) decided whether the beliefs represented a barrier or enabler to the implementation of the antenatal corticosteroid clinical practice guidelines. A frequency count was conducted on the data to illustrate the number of times a belief was mentioned within a particular behavioural domain. Where the word many or majority is used in the manuscript this refers to the frequency or strength of the belief among participants. Key domains to implementation of the New Zealand and Australian Antenatal Corticosteroid Clinical Practice Guideline were identified through consideration of: the presence of conflicting beliefs within a domain that would signal variation in provider attitudes and beliefs, the frequency of specific beliefs across transcripts and the likely impact of the belief on the behaviour [28]. Further analysis was performed to identify any differences in the enablers or barriers identified within the different health professional groups and sites that the participant worked at.

## Results

**Fig. 1:**
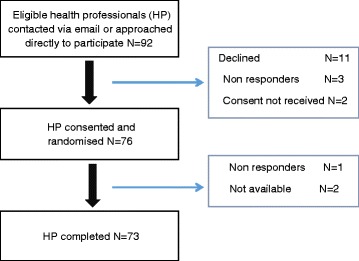
Flowchart of recruitment

A total of 73 health professionals completed either a semi-structured interview (*n* = 35) or online questionnaire (*n* = 38) (Fig. 1). Participants represented all four health professional groups and were predominantly female (78 %), aged between 40–59 years (66 %) and working within the hospital environment (89 %) (Table 1). Purposive sampling facilitated adequate sampling of health professionals of different ages, ethnicity and years working in the professional groups. Data saturation was reached among the different health professional groups and between hospital sites.

**Table 1:** Demographic characteristics of health professional participants (*n* = 73)

Characteristics of health professionals included in the study				
		Obstetrician (*n* = 25)	Neonatologist/paediatrician (*n* = 24)	Midwife (*n* = 24)
Age Group (years)	20–29	1	1	0
	30–39	8	6	3
	40–49	11	8	6
	50–59	5	6	13
	≥60	0	2	2
	Unknown	0	1	0
Ethnicity	European	16	14	21
	Maori	1	1	1
	Pacific peoples	1	0	0
	Asian	2	6	1
	Middle Eastern/Latin American/African	4	1	1
	other (unknown)	1 Indian/South African	1	0
	Did not answer	0	1	0
Primary place of work	Hospital	25	23	16
	Community	0	0	7
	Other	0	1	1
	Did not answer	0	1	0
No of years working Profession (years)	0–5 years	4	4	3
	6–10 years	5	5	3
	11–15 years	8	4	3
	>15 years	7	10	15
	Unknown	1	0	0

### Barriers and enablers

Overarching beliefs identified as enablers or barriers to implementation of the guideline related to seven key behavioural domains from the TDF for enablers and five behavioural domains for barriers. Throughout the results the enablers and barriers are presented separately within their corresponding behavioural domain.

A number of additional beliefs were identified within the other behavioural domains of the TDF (memory, attention and decision making, goals and skills) but it was felt that these beliefs/behaviours expressed would not significantly influence implementation of the clinical practice guidelines.

### Enablers to implementation of the Antenatal corticosteroid clinical practice guidelines

Eleven overarching beliefs were identified as enablers to implementation (Table 2) and are detailed below under the behavioural domain that they correspond to. The frequency or strength of the different beliefs identified among participants within the behavioural domains are detailed.

**Table 2:** Enablers to the implementation of the New Zealand and Australian Antenatal Corticosteroid Clinical Practice Guidelines

Corresponding behavioural domain within the TDF	Specific belief	Frequency
Belief about consequences	Use of the new antenatal corticosteroid clinical practice guideline will ensure optimum care for mothers and their babies	26
	Administration of antenatal corticosteroids is routine practice and improves outcomes	87
Knowledge	The evidence that supports the administration of antenatal corticosteroids is strong but I am aware of the gaps in the research	44
Social influences	Administration of antenatal corticosteroids is facilitated by discussion amongst the multidisciplinary team in conjunction with the woman	76
	Administration of antenatal corticosteroids is a social norm	25
Environmental context and resources	Antenatal corticosteroids are readily available and easy to administer.	29
	Adherence and use of Clinical practice guidelines is part of the organisational culture	23
Belief about capabilities	Prescription of antenatal corticosteroids is directed by senior obstetric health professionals	53
Social professional role and identity	Use of clinical practice guidelines helps to standardise practice and ensure consistency	25
	A new antenatal corticosteroid guideline will facilitate decision making	35
Behavioural Regulation	The guideline should be actively disseminated in a manageable format and include education and implementation resources	43

*Belief about the consequences* of antenatal corticosteroid administration, in improving health outcomes to infants, was high among study participants. A consistent comment across the participants was that administration of antenatal corticosteroids is routine practice and improves health outcomes, particularly in the administration of a single course of antenatal corticosteroids to women at risk of preterm birth. This is illustrated by the quote: “Too make sure that everyone who is eligible gets or receives them so the babies get the best deal”.

*Knowledge* of the evidence relating to antenatal corticosteroids varied among the health professional participants. Participants who were up to date in their knowledge of the evidence base, were much more positive in the belief that, overall, the evidence supported the administration of antenatal corticosteroids but were aware of the gaps in the research. Demonstrated by the quote: “So in the babies that we know from research will have benefits”.

When asked about *social influences*, participants expressed that administration of antenatal corticosteroids was considered a social norm within their organisations and that administration of antenatal corticosteroids was facilitated by discussion amongst the multidisciplinary team in conjunction with the woman demonstrated by the response: “I think the midwifery and obstetric staff are on the ball for the evidence regarding a single course”.

Participants believed that current *environmental context and resources* within their organisations facilitated the prescription and administration of antenatal corticosteroids. This is reflected by the comment: “There has not ever been a problem, doing that”.

When participants were asked about their *beliefs about capabilities* in the prescription and administration of antenatal corticosteroids a consistent comment was that prescription of antenatal corticosteroids was primarily believed to be directed by the senior obstetric health professionals within the organisation. When asked what difficulties or problems they had encountered in the prescription or administration of antenatal corticosteroids, the majority of health professionals responded that this decision was not made by them but was made by the obstetrician.

Use of clinical practice guidelines was identified as a professional standard. Within the behavioural domain of *social professional role and identity*, a consistent comment among participants was that use of clinical practice guidelines helps to standardise practice and ensure consistency. When asked what the purpose of the new antenatal corticosteroid clinical practice guideline should be participants felt it should be to facilitate decision making and to clarify uncertainties. Participants believed that a consequence of using the guideline ensured the provision of optimum care to mothers and their babies. This is illustrated by the quote: “To ensure best practice is consistent among all centres”.

Participants strongly believed that to facilitate *behavioural regulation* to encourage uptake and adherence to the recommendations within the new Antenatal Corticosteroid Clinical Practice Guideline, efforts should be made to actively disseminate the guideline. In response to the question; how do you think the guideline should best be implemented a consistent comment across the health professional participants was that the guideline should be actively disseminated in a manageable format and include education and implementation resources. Participants reported that interactive education sessions with worked examples would help them relate the recommendations to routine clinical encounters. Printed education materials and audit and feedback were identified as useful tools in facilitating ease of access and adherence to the new clinical practice guideline recommendations.

### Barriers to implementation of the antenatal corticosteroid clinical practice guidelines

Thirteen overarching beliefs were identified as barriers to implementation (Table 3) and are detailed below under the behavioural domain that they correspond to.

**Table 3:** Barriers to the implementation of the New Zealand and Australian Antenatal Corticosteroid Clinical Practice Guidelines

Corresponding behavioural domain within the TDF	Specific belief	Frequency
Belief about consequences	There is uncertainty around the use of antenatal corticosteroids at term and practice doesn’t necessarily reflect the evidence	25
	Use of antenatal corticosteroids improves outcomes of diabetic babies but their use in diabetic women can be difficult	16
	The use of repeat antenatal corticosteroids is known to be beneficial but concern exists around potential adverse effects.	41
Knowledge	My knowledge on the evidence related to antenatal corticosteroids is limited	50
	The evidence that supports the use of repeat antenatal corticosteroids is conflicting	37
	I need more clarification on the evidence regarding antenatal corticosteroid administration in specific populations	29
	There is confusion in antenatal corticosteroid practice in the understanding of a course, dose and duration between doses and courses.	14
	My understanding of antenatal corticosteroids comes from what I witness in clinical practice	6
Social influences	Lack of consistency and difference of opinion make it difficult to know what is correct antenatal corticosteroid practice	48
Environmental context and resources	Competing tasks and time constraints impact on antenatal corticosteroid administration	12
	Ease of access, readability and implementation tools/education discourages/encourages use of guidelines	60
Social professional role and identity	My use of the guideline would be dependent on it being based on good evidence	8
	Clinical practice guidelines assist in decision making but often clinical judgement supersedes this	22

*SMO* senior medical officer, *DHB* district health board

Within the domain *environmental context and resources*, a consistent comment across participants was that ease of access to the clinical practice guideline within the clinical environment will strongly influence its use. Many reported difficulties with current clinical practice guidelines relating to the ability to access them easily. Another consistent statement was that consideration should be given to the readability and format of the guideline. Many expressed the belief that if a guideline was too long participants would be reluctant to read it. This is illustrated by the comment: “if they are too wordy people don’t read them”. Participants reported that time constraints both in the acute nature of the situation that antenatal corticosteroids are often prescribed and the intensity of the work load in the obstetric environment often hinder antenatal corticosteroid administration.

Limited *knowledge*, outdated knowledge and uncertainties in the evidence, represented a barrier for some health professionals. In some instances health professionals reported their knowledge comes from what is witnessed in clinical practice rather than knowledge of the evidence as reflected by the comment: “I know it is starting to come into practice. But I am not aware of the research on that”. There was confusion in some of the terminology related to antenatal corticosteroid use including the understanding of a course, dose and duration between doses and courses. This is shown by the statement: “Single dose. You mean the following doses, the weekly”. One of the most significant barriers identified was within the domain of *social influences*. The belief that lack of consistency and difference of opinion between individual health professionals makes it very difficult to know what correct or standard antenatal corticosteroid practice is. This was particularly relevant for midwives and more junior members of the health care team, illustrated by the statement: “Even within institutions like this there will be people with different opinions. That is one of the problems in an institution like this that there are lots of different opinions”.

Participants were mixed in their *beliefs about the consequences* of using antenatal corticosteroids. Whilst participants believed in the efficacy of antenatal corticosteroids many remain uncertain about their use in specific populations including the use of repeat antenatal corticosteroids, the use of antenatal corticosteroids at term gestations and antenatal corticosteroid administration in high risk groups, particularly in women with diabetes. There was some concern that currently practice doesn’t necessarily reflect the evidence regarding the administration of antenatal corticosteroids at term. This is reflected by the comments: “There is a little less certainty about them, you know about the benefits of repeat corticosteroids and of course the possible side effects on growth” and “Before caesarean section, well the evidence is limited but we are doing it”.

Whilst the majority of participants within the domain of *social professional role and identity*, reported the use of clinical practice guidelines as being a professional standard, a number of participants voiced some scepticism on the evidence used to create guidelines and that this could potentially impact on their use. This is demonstrated by the statement: “Well I would like them to be based on evidence”. A proportion of participants expressed the belief that guidelines assist in their decision making but may not determine the clinical decisions that they make as seen by the comment: “Sometimes things may deviate from the normal and another clinical decision may need to be made in the best interest of the patient”.

### Differences across health professional groups

Despite the overarching barriers and enablers demonstrated across the health professional groups, occasionally the different health professional groups beliefs within a behavioural domain were opposing. In addition there were some additional beliefs identified between individual health professional groups within behavioural domains (Table 4).

**Table 4:** Different beliefs identified within behavioural domains between individual health professional groups

Behavioural domain	HP Group	Different specific beliefs within a domain	Sample quote from health professional group	B/E	^a^Frequency
Belief about consequences	Neo	The evidence suggests administering a repeat course/(s) of antenatal corticosteroids is beneficial	“*I think people are usually confident that they are not causing harm in the lower number*”	E	18
	Neo	I do not believe the evidence suggests administering antenatal corticosteroids at term is beneficial	“*There is limited evidence. I mean it sort of makes sense. But then you have to start thinking about how you prime corticosteroid receptors in later life*”	E	12
	Neo	The latest gestational age I would consider administering antenatal corticosteroids would be 34 weeks	“*34 weeks as being the typical break point and I think that is a sensible break point based on the current evidence*”	E	10
	Obs	The latest gestational age I would consider administering antenatal corticosteroids would be up to 37/38 weeks	“*Well I guess for*, *for our elective caesareans we have done if we have delivered them less than 39 weeks*”	E	13
Social professional role and identity	Mw	Having knowledge on the administration of antenatal corticosteroids is not required by my professional body	“*As a professional group the college of midwives want us to be grounded in the normal*”	B	3
			“*Is just out of intellectual interest rather than it necessarily being something that I need to know for what I am actually practising*”		
	Neo	The neonatal team confirm antenatal corticosteroids have been administered to the appropriate women	“*I don*’*t prescribe antenatal corticosteroids. I often ask if they have been prescribed*”	E	7
	Neo	Neonatologists advise on antenatal corticosteroid administration at extremes of viability	“*Due to poor prognosis in less than 24*/*40 do not feel giving steroids to be appropriate*”	E	3
Environmental context and resources	Obs	Further guidelines and protocols are needed to guide use of antenatal corticosteroids	“*Facilitate primary course*, *confusion regarding secondary course*” (*do external influences facilitate or hinder the use of ACS*)	B	3
Emotion	Obs	I find discussions around viability quite difficult	“*If you ask me personally what I would do if it was me*, *that*’*s a tough decision. The query viability stuff is no easy street*”	E	3
	Mw	Overloading patients with information around antenatal corticosteroids could scare or confuse them.	“*Because you don*’*t want to frighten the life out of them*” (*informing patients about steroids*)	E	2
	Neo	I am frustrated by some elements of antenatal corticosteroid practice amongst obstetricians and the poor communication with the neonatal team	“*Obstetric staff to think of this when prescribing the first dose and counselling patients accordingly*”	B	5
	Obs	I am frustrated by the conflicting information and practice around repeat antenatal corticosteroid administration	“*To be honest I don*’*t know the evidence of this whole repeat and rescues and things and it would be good to have that simplified and easy to access*”	B	2

*HP group* health professional group, *obs* obstetrician, *Neo* neonatologist/paediatrician, *mw* midwife, *B* barrier to implementation of the new antenatal corticosteroid clinical practice guidelines, *E* enabler to implementation of the new antenatal corticosteroid clinical practice guidelines, *ACS* antenatal corticosteroids

^**a**^Frequency of specific beliefs within a behavioural domain

Within the domain *belief about consequences*, uncertainty in the evidence base related to antenatal corticosteroids in specific groups including, the use of repeat antenatal corticosteroids, the use of antenatal corticosteroids at term gestations and antenatal corticosteroid administration in high risk groups, particularly in women with diabetes was demonstrated across all participants. However, it was evident that the different health professional groups varied in their interpretation of the evidence related to antenatal corticosteroids. Neonatologists were much more certain that the evidence suggested that prescribing a repeat course/(s) of antenatal corticosteroids was beneficial compared to obstetric and midwifery participants. This is demonstrated by the responses: “I think people are usually confident that they are not causing harm in the lower number (related to administration of antenatal corticosteroids) (Neonatologist/Paediatrician) compared to: “Repeat course is controversial, I will say that,…first do no harm. So I have seen it that sometimes it is taken lightly the decision to do repeat doses” (Obstetrician). Furthermore obstetricians were more likely to believe that administration of antenatal corticosteroids at term was beneficial, compared with both the neonatologists and midwives who predominantly felt that the evidence did not suggest this to be the case. Reflected by a Neonatologist/Paediatrician comment: “There is limited evidence. I mean it sort of makes sense but then you have to start thinking about how you prime corticosteroid receptors in later life” compared to an Obstetrician comment: “So that we have said if hmm, if it is an elective caesarean section because the risks of RDS exist. Even they are low. Perhaps doing steroids which we feel do not have any long lasting ill health or anything you might want to call it. Perhaps it is a reasonable thing to do”.

These differences in beliefs were also reflected when health professionals were asked the latest gestational age they would administer antenatal corticosteroids too. Neonatologists felt that the latest gestational age they would consider administering antenatal corticosteroids to would be 34 weeks’ gestation, whereas obstetricians would consider administering up to term gestations’ in particular instances.

Despite the overarching belief within the domain *social professional role and identity*, that prescription of antenatal corticosteroids is directed by senior obstetric health professionals, the various health professional groups viewed their professional roles and identity relating to antenatal corticosteroids differently. Neonatologists felt that their role was in confirming antenatal corticosteroids had been administered to the appropriate women and that they should be actively involved in discussions with women, their families and the lead maternity carer around the administration of antenatal corticosteroids at extremes of viability. Midwifery participants were mixed in their beliefs about their role in the prescription and administration of antenatal corticosteroids. A number of midwives were unsure whether having knowledge on the administration of antenatal corticosteroids was required within their scope of practice. This is demonstrated by the comment: “It is just out of intellectual interest rather than it necessarily being something that I need to know for what I am actually practising”. A significant proportion of the midwifery, obstetric and neonatal participants felt that midwives provided a very significant role in facilitating the prescription and administration of antenatal corticosteroids reflected by the comment: “we have come in we have figured out what is going on they have been given steroids they been given nifedipine if that was supposed to happen”.

An additional belief within *environmental context and resources*, demonstrated by obstetric participants was that they reported the urgent need for guidelines to address some of their clinical uncertainties. This was particularly related to the use of antenatal corticosteroids in specific obstetric populations (women with diabetes, women undergoing a caesarean section) and related to the gestational age of administration.

*Emotion* was demonstrated across the health professional groups but different emotions were identified among the different health professional groups and related to varying aspects of antenatal corticosteroids prescription and administration. Obstetricians demonstrated vulnerability when asked what the earliest gestational age they would administer antenatal corticosteroids to, reporting that they often found this quite a difficult conversation with the women and their family. This is reflected by the comment: “If you ask me personally what I would do if it was me that’s a tough decision. The query viability stuff is no easy street” (Obstetrician). Both obstetric and neonatology participants demonstrated frustration related to different aspects of antenatal corticosteroid prescription and administration. Neonatology participants reported some frustration related to obstetric practice around antenatal corticosteroids particularly related to poor communication with the neonatal team as illustrated by the comment: “what happens is that we are lucky if they actually say they have had a course of steroids” (Neonatologist/Paediatrician): Obstetricians demonstrated frustration in relation to their perceived uncertainties around the evidence related to the prescription and administration of repeat course/s of antenatal corticosteroids. Midwives reported concern around overloading and confusing women with information related to antenatal corticosteroid administration as reflected by the comment: “Because you don’t want to frighten the life out of them” (informing patients about antenatal corticosteroids) (Midwife).

### Differences across organisations

Although there were significant overarching barriers and enablers demonstrated across the three district health boards there were also some distinct differences identified. Within the three domains; *belief about consequences*; *belief about capabilities* and *social influences* there were some key organisational differences identified. In particular interpretation of the evidence and confidence in prescribing or administering antenatal corticosteroids varied depending on the organisation. The strength and presence of certain behaviours were identified at some sites and not at others (Table 5).

**Table 5:** Different beliefs identified within behavioural domains between health care organisations

Behavioural domain	Organisation	Different beliefs within a domain	Barrier/Enabler (B/E) example statement	B/E	^a^Frequency
Belief about consequences	No 3	I am uncertain about whether the evidence suggests prescribing repeat course/(s) of antenatal corticosteroids is beneficial	“*I think there seems to be some variation amongst clinicians as to whether they think the repeat doses are actually beneficial for the infant and I think my understanding of the literature is that they do reduce the severity of respiratory distress*” *Or* “*I am not to convinced about that if we*, *we should be doing routinely repeat*”	B	7
	No 2	Use of repeat antenatal corticosteroids is beneficial	“*My sort of era were taught by professor liggins to give them every 2 weeks. And So that*’*s what we have done and so we haven*’*t usually had to give more than 2 or 3 doses*.”	E	8
	No 2	I do not believe the evidence suggests administering antenatal corticosteroids at term is beneficial	“*No I don*’*t prescribe to that and I don*’*t think that they are warranted*” (*steroids at term*)	E	12
	No 3	Use of antenatal corticosteroids at term is beneficial	“*So that we have said if hmm*, *if it is an elective caesarean section because the risks of RDS exist. Even they are low. Perhaps doing steroids which we feel do not have any long lasting Hmm ill health or anything you might want to call it. Hmm*, *perhaps is a reasonable thing to do*”	B	9
Belief about capabilities	No 3	I am unsure about prescribing or administering repeat antenatal corticosteroids to diabetic women who remain at risk of preterm birth	“*So you know they weigh it up a bit more with the diabetics. Hmm*, *repeats not so much in the diabetic population*”	B	12
	No 1	Use of antenatal corticosteroids improves outcomes of diabetic babies	“*I would feel more comfortable doing that and having terrible sugars for 24 or 48 h or however long that would be because our physicians would manage that*”	E	10
Social influences	No 1	Deciding to administer antenatal corticosteroids is a joint decision between obstetrics and neonatology	“*that*’*s a relationship between neonatologists and obstetricians*” or “*only be after significant input from paediatrics*”	E	10
	No 2	I am not involved in making decisions on antenatal corticosteroid administration	“*No but I am distanced from the Obstetric management*”	B	5

*B* barrier to implementation of the new antenatal corticosteroid clinical practice guidelines, *E* enabler to implementation of the new antenatal corticosteroid clinical practice guidelines, *RDS* respiratory distress syndrome

^**a**^Frequency of specific beliefs within a behavioural domain

*Belief about consequences* in administering repeat course (s) or doses, and in the administration of antenatal corticosteroids at term varied between the different organisations. Whilst administration of antenatal corticosteroids was an inherent part of routine practice in one organisation, it was still viewed with caution in another. This is reflected by the comments: “I am not too convinced about that, if we should be routinely doing repeat…” (organisation number 3) compared to: “My sort of era we were taught by Professor Liggins to give them every 2 weeks, and so that is what we have done” (organisation number 2). Similarly there was significant variation in practice between organisations in whether or not antenatal corticosteroids were routinely being given for women undergoing elective caesarean section at term.

*Beliefs about capabilities* in administering antenatal corticosteroids to women considered high risk including; women with diabetes, pre-eclampsia or who had experienced preterm prelabour rupture of membranes varied considerably between the three organisations. This is illustrated by the comments: “So you know they weigh it up a bit more with diabetics. Hmm, repeats no so much in the diabetic population (organisation number 3) compared to: “I would feel more comfortable doing that and having terrible sugars for 24 or 48 h or however long that would be because our physicians would manage that” (organisation number 1).

Within the domain of *social influences*, the degree of interdisciplinary decision making on whether antenatal corticosteroids should be administered varied significantly between the organisations. One organisation reported that administration of antenatal corticosteroids was considered a joint decision between obstetrics and neonatology reflected by the comment: “only after significant input from paediatrics” (organisation number 1) whereas the other organisations felt in was very much a role restricted to the obstetricians, demonstrated by the comment “No but I am distanced from obstetric management” (Organisation number 3).

## Discussion

Our study demonstrated that the validated TDF could provide a useful framework to guide an assessment of potential barriers and enablers to implementation of the New Zealand and Australian Antenatal Corticosteroid Clinical Practice Guidelines. Analysis demonstrated that a number of behaviours need to be changed or addressed to ensure successful implementation of the guideline. Overarching beliefs that might pose as barriers to implementation of the guideline were identified within the five domains of: *environmental context and resources*; *knowledge*; *social influences*; *belief about consequences and social professional role and identity*.

The identification of significant uncertainties and variation in prescribing practice of antenatal corticosteroids has been demonstrated both nationally and internationally [15, 29]. It would appear that the variation may be attributed to evidence practice gaps with a proportion of the health professionals participating in the study being unaware of the current evidence base. Other participants however, seemed to struggle to draw conclusions on best practice due to difficulties in accessing or synthesizing the large volumes of evidence that exist on antenatal corticosteroids and were unaware of currently available systematic reviews that may guide them [14, 30–32]. The new clinical practice guidelines should address some of these clinical uncertainties if the recommendations are accessed, accepted and adopted into practice. Additionally, on occasions health professionals demonstrated an inherent scepticism towards clinical practice guidelines, particularly in relation to the source of the evidence and the processes involved in synthesizing the evidence and generating recommendations. Techniques such as information provision and persuasive communication could be facilitated by workshops to inform or update health professionals on the synthesised evidence and subsequent recommendations related to antenatal corticosteroids. This could include detail of the methodological rigour used to develop the guidelines.

The variation in interpretation of the evidence demonstrated between the individual health professional groups potentially identifies the need for any interventions to include all members of the multi-disciplinary team to accommodate discussion of the evidence and standardisation of practice. Academic meetings could facilitate clarification of individual health professional roles and responsibilities, as perceived by the individual group and their peers in the prescription and administration of antenatal corticosteroids. Multidisciplinary meetings could encourage improved interaction between the different health professional groups and help redefine roles, responsibilities and engagement of the different health professionals involved and modify the barriers identified within the behavioural domains of social professional roles and identity and social influences.

We have explored the influence of the different organisations on the barriers and enablers identified. Variation in beliefs about the evidence appeared to reflect the different prescribing practice within the organisations. These findings have potential implications on implementation particularly in addressing whether intervention components can be generalisable or need to be site specific [1]. It is apparent that some organisations will need to modify or change their current practices related to antenatal corticosteroid administration to a greater degree to ensure adoption of the new clinical practice recommendations. Resulting in the question of, whether more intensive or site specific interventions are required to reflect local contexts. Furthermore if this variation in practice is reflective of organisations throughout the rest of Australia and New Zealand, this would suggest that the recommendations proposed in the new antenatal corticosteroid guideline needs to be presented at academic meetings locally, nationally and internationally across both countries to facilitate knowledge and uptake of the recommendations within the clinical practice guidelines.

This study identified elements related to environmental context and resources that impact on health professionals’ prescription and administration of antenatal corticosteroids. Participants often reported that time constraints hinder access and use of clinical practice guidelines, and the prescription and administration of antenatal corticosteroids. The specific nature of the obstetric environment is thought to influence the success of specific implementation interventions [33]. Due to the nature of the care provided, which is both preventative and curative, the strong medico-legal concerns associated with obstetrics and often the short decision time encountered in emergency situations, many participants in our study expressed the need for the guidelines to be easily accessible and accompanied by multifaceted intervention tools. Through the process of this study we were able to examine some of the principles of reliability science [34] by asking health professionals to identify implementation tools that would facilitate uptake of the guideline recommendations and subsequently improve practice. Participants expressed the need for implementation tools that facilitate prescription and administration of antenatal corticosteroids and enable monitoring of practice over time to ensure compliance with the guideline recommendations.

Health professionals identified specific implementation interventions that they believed would be most useful including; education sessions [35], audit and feedback [20] and printed educational resources [18]. A similar study by the World Health Organisation identifying barriers and facilitators towards implementing guidelines to reduce caesarean section identified audit and feedback as a useful tool in facilitating change in practice [36]. The potential mechanism of action proposed was awareness of local performance against explicit criteria acted as the key stimulus to change practice.

Thematic analysis has identified overarching beliefs within seven key behavioural domains that could facilitate implementation of the guideline. The overarching beliefs in the efficacy of antenatal corticosteroids and the value of clinical practice guidelines in improving health outcomes could be used in education workshops to encourage professionals to modify their practice in line with the new guideline recommendations. Clinicians report using guidelines to validate their decision making. Therefore reported difficulties in prescribing antenatal corticosteroids to high risk groups or repeat dose/(s) of antenatal corticosteroids could be addressed with techniques including practical or on-line sessions with patient vignettes to demonstrate use of guideline recommendations [37–39]. Identification of obstetricians as leaders in the prescription and administration of antenatal steroids suggest that experts within obstetrics who practice in accordance with the clinical practice recommendations could be used as expert opinion leaders to demonstrate appropriate use of the guideline [21].

In order to increase the likelihood of success of uptake of guideline recommendations into routine practice researchers in implementation science are attempting to map interventions too pre-identified behavioural determinants [8, 40–42]. This study has identified behavioural determinants that interventions can be modelled against in an attempt to overcome modifiable barriers and enhance the enablers [43]. Our study suggests that a complex intervention strategy inclusive of techniques such as persuasive communication, rehearsal of behaviour and demonstration of the required behaviour by a peer expert with a multidisciplinary approach could lead to successful implementation of the new antenatal corticosteroid guideline across Australia and New Zealand. This is one of the few studies that has explored the influence of the different health professional groups and the organisation on the barriers and enablers identified.

Our study is limited by restriction to only one geographical area in a single country. However the overarching barriers and enablers were demonstrated across all three of the organisations. The sample size was sufficient to facilitate further analysis to be undertaken to explore the influence of different health professional groups and organisations on the barriers and enablers identified.

The results of our study may be limited by the fact that a significant proportion of the health professionals volunteered to take part. Individuals who took part may have a higher degree of interest in clinical practice guidelines and in antenatal corticosteroids compared with other health professionals. However the sample size and purposeful sample method ensured that the participants were likely to be representative of the organisations involved in the study. In addition a key strength of this study is that it was conducted prior to implementation of the new antenatal corticosteroid guideline to identify potential barriers and enablers to implementation to be addressed or built upon.

Conducting the study on a larger scale across Australia and New Zealand could potentially identify other barriers or enablers to implementation of the new antenatal corticosteroid guideline and may provide a greater understanding of the issues raised in our study. In addition it would be useful to conduct further research on other guidelines within the same cohort of participants to see if the barriers and enablers are transferable.

## Conclusions

Our study has identified the barriers and enablers to implementation of the New Zealand and Australian Antenatal Corticosteroid Clinical Practice Guidelines, as perceived by New Zealand health care professionals. Despite the overarching barriers and enablers demonstrated across health professional groups and organisations, there were some additional beliefs and contrary beliefs identified between the different health professional groups and the organisations. The study findings will be used to model intervention tools to address the identified barriers and enhance the recognised enablers. This should facilitate process evaluations to improve understanding on how and why any change interventions are successful.

## Additional file

Additional file 1: Questions for semi-structured interviews and online questionnaires and their corresponding theoretical domain. (DOCX 13 kb)

## Abbreviations

ACS: Antenatal corticosteroids
B: Barrier to implementation of the new antenatal corticosteroid clinical practice guidelines
DHB: District health board
E: Enabler to implementation of the new antenatal corticosteroid clinical practice guidelines
HP group: Health professional group
Mw: Midwife
Neo: Neonatologist/paediatrician
Obs: Obstetrician
RDS: Respiratory distress syndrome
SMO: Senior medical officer
TDF: Theoretical domains framework
