# Supplementary material for: Identifying the barriers and enablers in the implementation of the New Zealand and Australian Antenatal Corticosteroid Clinical Practice Guidelines
Source: BMC Health Serv Res. 2016 Oct 28;16:617. doi: 10.1186/s12913-016-1858-8 (PMC5084422; doi:10.1186/s12913-016-1858-8)
Supplement: Additional file 1: — Questions for semi-structured interviews and online questionnaires and their corresponding theoretical domain. (DOCX 13 kb) [file 12913_2016_1858_MOESM1_ESM.docx]

Questions for semi-structured interviews and online questionnaires and their corresponding theoretical domain.

| Domains | Interview Questions |
| --- | --- |
| Knowledge | |
|  | Are you aware there is a new binational antenatal corticosteroid guideline being produced |
|  | Do you think the guideline needs to give a recommendation on the use of a single course of antenatal corticosteroids for preterm birth |
|  | Do you think the guideline needs to give a recommendation on the use of repeat course/s of antenatal corticosteroids for preterm birth |
|  | Do you think the guideline needs to clarify the evidence on the use of antenatal corticosteroids at term |
|  | Which antenatal corticosteroid do you use in your unit/practice |
|  | What dose do you prescribe or administer for a single course of ACS |
|  | What dose do you prescribe or administer for a repeat course of ACS |
| Skills | |
|  | How confident are you in prescribing a single course of antenatal corticosteroids |
|  | How confident are you in prescribing repeat courses of antenatal corticosteroids |
|  | How many courses would you be happy prescribing |
|  | Are you confident prescribing corticosteroids to high risk groups including: diabetic women, pre-eclamptic women, women with preterm prelabour rupture of membranes |
|  | Would you prescribe repeat antenatal corticosteroids for these groups if they remained at risk of preterm birth |
| Social professional role and identity | |
|  | Do you think clinical practice guidelines should influence your behaviour in the clinical setting |
| Belief about capabilities | |
|  | What difficulties/problems have you personally encountered in prescribing/administering a single course of antenatal corticosteroids |
|  | What difficulties/problems have you personally encountered in prescribing/administering a repeat course of antenatal corticosteroids |
|  | Are there any instances where you would not or have witnessed others not prescribe antenatal corticosteroids |
| Intentions | |
|  | what are your current motivations to prescribing antenatal corticosteroids |
| Memory, attention and decision making | |
|  | Do you always remember to prescribe single/repeat antenatal corticosteroids |
| Environmental context and resources | |
|  | Do resources facilitate the prescription of antenatal corticosteroids |
|  | Do you feel there are competing tasks/time constraints to prescribing antenatal corticosteroids |
|  | Are the necessary resources/systems in place in your birthing unit to allow you to prescribe antenatal corticosteroids |
| Social influences | |
|  | Do external influences (eg, other Consultants/Professional groups) facilitate or hinder prescription/administration of antenatal corticosteroids? |
|  | Are there specific persons/professional groups you would take your lead from |
| Emotion | |
|  | Do emotional issues/factors facilitate or hinder your use of antenatal corticosteroids |
| Behavioural regulation | |
|  | In your opinion what steps are needed to enable the uptake and use of the recommendations within the antenatal corticosteroid guideline |
|  | How do you think the antenatal corticosteroid guideline could best be implemented |
| Belief about consequences | |
|  | Overall, do you feel the evidence that you are currently aware of suggests that prescribing antenatal corticosteroids is beneficial: for a single course of antenatal corticosteroids, for repeat courses and for ACS prescribed at term |
|  | How comfortable do you feel NOT prescribing a single/repeat course of antenatal corticosteroids |
|  | Do you find prescribing antenatal corticosteroids at very early gestations eg, 23/40 compatible with your own professional standards and beliefs |
|  | What is the earliest gestational age you would prescribe antenatal corticosteroids |
|  | What is the latest gestational age you would prescribe antenatal corticosteroids |
| Reinforcement | |
|  | What would help in your clinical practice to make it easier for you to prescribe/administer a single course of antenatal corticosteroids (eg, prompts, alerts) |
|  | What would help in your clinical practice to make it easier for you to prescribe/administer a repeat course of antenatal corticosteroids (eg, prompts, alerts) |
| Optimism | |
|  | How important do you think antenatal corticosteroids are in the care of preterm infants |
| Goals | |
|  | What do you think the purpose of the new antenatal corticosteroid guideline should be |

ACS: antenatal corticosteroids.
